# Altered Variability and Concordance of Dynamic Resting-State fMRI Indices in Patients With Attention Deficit Hyperactivity Disorder

**DOI:** 10.3389/fnins.2021.731596

**Published:** 2021-09-16

**Authors:** Feiling Lou, Jiejie Tao, Ronghui Zhou, Shuangli Chen, Andan Qian, Chuang Yang, Xiangwu Zheng, Bicheng Chen, Zhishan Hu, Meihao Wang

**Affiliations:** ^1^Department of Radiology, The First Affiliated Hospital of Wenzhou Medical University, Wenzhou, China; ^2^Department of Psychiatry, The First Affiliated Hospital of Wenzhou Medical University, Wenzhou, China; ^3^Key Laboratory of Diagnosis and Treatment of Severe Hepato-Pancreatic Diseases of Zhejiang Province, Zhejiang Provincial Top Key Discipline in Surgery, The First Affiliated Hospital of Wenzhou Medical University, Wenzhou, China; ^4^State Key Laboratory of Cognitive Neuroscience and Learning, Beijing Normal University, Beijing, China

**Keywords:** ADHD, resting-state fMRI, temporal dynamics, concordance, variability

## Abstract

**Objective:** Attention deficit hyperactivity disorder (ADHD) is a commonly diagnosed neuropsychiatric disorder in children, which is characterized by inattention, hyperactivity and impulsivity. Using resting-state functional magnetic resonance imaging (R-fMRI), the alterations of static and dynamic characteristics of intrinsic brain activity have been identified in patients with ADHD. Yet, it remains unclear whether the concordance among indices of dynamic R-fMRI is altered in ADHD.

**Methods:** R-fMRI scans obtained from 50 patients with ADHD and 28 healthy controls (HC) were used for the current study. We calculated the regional dynamic changes in brain activity indices using the sliding-window method and compared the differences in variability of these indices between ADHD patients and HCs. Further, the concordance among these dynamic indices was calculated and compared. Finally, the relationship between variability/concordance of these indices and ADHD-relevant clinical test scores was investigated.

**Results:** Patients with ADHD showed decreased variability of dynamic amplitude of low-frequency fluctuation (dALFF) in the left middle frontal gyrus and increased one in right middle occipital gyrus, as compared with the HCs. Besides, ADHD patients showed decreased voxel-wise concordance in the left middle frontal gyrus. Further, lower voxel-wise concordance in ADHD’s left middle frontal gyrus was associated with more non-perseverative errors in Wisconsin Card Sorting Test, which reflects worse cognitive control.

**Conclusion:** Our findings suggest that variability and concordance in dynamic brain activity may serve as biomarkers for the diagnosis of ADHD. Further, the decreased voxel-wise concordance is associated with deficit in cognitive control in ADHD patients.

## Introduction

Attention deficit hyperactivity disorder (ADHD) is characterized by inattention, hyperactivity and impulsivity. It is one of the most commonly diagnosed childhood neurodevelopmental disorder, whose prevalence in school-age children and adolescents is estimated at 7.2% (Thomas et al., 2015). This disorder seriously affects these children’s academic performance and social skills and causes immense burden for their families.

Maladaptive in the intrinsic brain activity of ADHD was frequently reported in previous studies. Many of these findings were derived from resting-state functional magnetic resonance imaging (R-fMRI), which measures the blood oxygen level dependent (BOLD) signal without task or stimulation and further characterizes the spatiotemporal organization of brain activities (Forster et al., 1998; Fox and Raichle, 2007). In specific, multiple R-fMRI indices have been developed to depict the intrinsic BOLD signal. For example, amplitude of low frequency fluctuation (ALFF) characterizes the intensity of local activity of brain spontaneous activity (Zang et al., 2007; Zou et al., 2008); Regional homogeneity (ReHo) measures the consistency of regional neural activity (Zang et al., 2004); Degree centrality (DC) describes the association between a local area with the whole brain (Buckner et al., 2009; Zuo et al., 2012); Voxel mirror homotopic connectivity (VMHC) represents the functional connectivity between each pair of symmetrical interhemispheric voxels (Zuo et al., 2010). Global signal connectivity (GSC) is described as the correlation between local voxels and global signal (Hahamy et al., 2014; Power et al., 2017).

These R-fMRI indices have been used to characterize the maladaptive brain functions in patients with ADHD. For example, children with ADHD exhibited increased ALFF in the occipital cortex and globus pallidus (An et al., 2013; Li et al., 2014), and decreased one in the left orbitofrontal cortex and ventral superior frontal gyrus (Li et al., 2014), showing hyperactive visual processing and hypoactive frontal control. Meanwhile, increased ReHo was reported in the bilateral lingual and fusiform gyri and right dorsal anterior cingulate cortices, and decreased ReHo in the bilateral ventral medial prefrontal cortex, cerebellum and right middle frontal gyrus, and right precuneus (An et al., 2013), which again indicated deficit in frontal lobe. In addition, the increased ReHo in right dorsal anterior cingulate cortex and left lingual gyrus, and the decreased ReHo in right cerebellum were associated with higher ADHD symptom scores in the ADHD group (An et al., 2013). Meantime, decreased DC was observed in the head of the right pallidum, caudate and putamen and in the left postcentral cortex (Di Martino et al., 2013), as well as right posterior cingulate gyrus and frontoparietal network (Jiang et al., 2019). Also, it was found that children with ADHD exhibited increased DC in the cerebellar anterior lobe, cingulate gyrus and middle occipital cortex, and increased VMHC in bilateral superior frontal, middle occipital, and cerebellar anterior lobes (Jiang et al., 2019). The findings above have proven that the R-fMRI indices can successfully capture the dysfunctions in the brains of ADHD patients.

Although these studies provided insights into the neural basis of ADHD, they failed to capture the dynamic changes in brain activities. Recent evidence demonstrated the temporal dynamic in brain activity patterns (Hutchison et al., 2013; Allen et al., 2014) and found that the alterations in these patterns were associated with neurological and psychiatric disorders. For example, patients with generalized anxiety disorder (GAD) exhibited increased variability in dynamic ALFF (dALFF) across wide spread brain regions (Cui et al., 2020). Meanwhile, patients with major depressive disorder (MDD) showed decreased variability in dALFF in the emotion network (Li et al., 2019). As for patients with ADHD, one study reported that they showed decreased mean value and variability in dynamic ReHo in the parietal lobe, compared to the healthy control group (Kim et al., 2018).

In addition, the concordance, i.e., temporal and spatial coupling, among multiple dynamic brain activity indices shows stable individual difference (Yan et al., 2017). Studies revealed that patients with psychiatric disorders exhibited decreased concordance in brain activity indices. For example, patients with schizophrenia showed decreased volume-wise concordance in whole gray matter and reduced voxel-wise concordance in multiple brain regions. Moreover, these concordance changes were negatively correlated with their onset age (Zhu et al., 2018). For MDD patients, reduced concordance was found in a wide range of brain regions, and the reduction was associated with impaired prospective memory and sustained attention (Zhu et al., 2019). Considering the tight relationship between the concordance of R-fMRI indices and mental disorders, the concordance may also underpin the ADHD. However, little has been done to explore this potential biomarker.

The current study aimed to bridge this gap. In specific, we compared the variability of dynamic R-fMRI indices and concordance among them between patients with ADHD and healthy controls, and further correlated the variability/concordance with the clinical test scores. Based on the results of previous studies, we hypothesized that ADHD patients would exhibit abnormalities in dynamic brain activity and its concordance, and this concordance would be related to its clinical manifestations. Our study may enhance the understanding of the neural mechanism underlying ADHD.

## Materials and Methods

### Participants

Fifty-eight drug-naïve ADHD patients were recruited from the Out-patient Clinic of the First Affiliated Hospital of Wenzhou Medical University. Meanwhile, 31 healthy controls matched in gender, age and IQ were recruited from local schools. The diagnosis of ADHD was performed by three experienced psychiatrists according to the Fifth Edition of the Diagnostic and Statistical Manual of Mental Disorders (DSM-V). For all the participants, the exclusion criteria were: (1) conduct disorder, oppositional defiant disorder, or any other psychotropic disorder besides ADHD; (2) history of major neurological or physical illness; (3) current or history use of psychotropic drugs; (4) intelligence quotient (IQ) lower than 80; (5) MR scanning contraindications. This study was approved by the Ethics Committee of the First Affiliated Hospital of Wenzhou Medical University. All participants were well informed and written informed consent was obtained before the test.

### Behavior and Cognition Assessment

The revised version of Conners’ Parent Rating Scale (CPRS) (Conners et al., 1998) was used to assess children’s behavioral problems, including conduct problems, study problems, psychosomatic disorders, impulse hyperactivity, anxiety, and hyperactivity.

The computerized Wisconsin Card Sorting Test (WCST) (Feldstein et al., 1999) was used to assess the executive function, including cognitive flexibility, working memory, continuous attention, and response inhibition. In specific, patients were required to match two cards according to the color, shape or quantity. After 10 trials of correct matching, one completed session was counted and the rule would be changed. The task was ended if the participants complete six sessions or 128 trials. The perseverative errors (at least two successive errors) and non-perseverative errors were counted. Note that non-perseverative errors were counted as the difference between the total number of errors and the number of perseverative errors.

### MRI Data Acquisition

Images were acquired in a 3T GE Sigma HDX 3.0 scanner (GE Healthcare, United States) with the following parameters: repetition time (TR) = 2,000 ms, echo time (TE) = 30 ms, 31 axial slices, slice thickness = 4 mm, slice gap = 0.2 mm, flip angle = 90°, matrix size = 64 × 64, field of view (FOV) = 192 × 192 mm, and 240 volumes. High-spatial-resolution T1-weighted 3D images were obtained axially using the following parameters:162 axial slices, repetition time (TR) = 7,200 ms, echo time (TE) = 2.2 ms, flip angle = 7°, section thickness = 1 mm, gap = 0, field of view (FOV) = 256 × 256 mm, data matrix = 256 × 256. During the scanning, sponge earplugs were used to reduce scanner noise, and foam cushions were used to minimize head movement. The subjects were required to be relax, stay still, close their eyes, and stay awake.

### Data Preprocessing

Data Processing and Analysis for Brain Imaging (DPABI) (Yan et al., 2016) was used for data preprocessing: (1) the first 10 volumes were eliminated to enable subjects to adapt to the scanning environment and the magnetic field to be stable; (2) slice timing correction; (3) head motion correction (subjects were excluded from further analysis if their maximal head motion exceeded 3 mm displacement or 3°of rotation, 8 ADHD patients and 3 HCs were therefore excluded, leaving 50 ADHD patients and 28 HCs); (4) structural image alignment, aligning the individual T1-weighted image to the average functional image through six-degree-of-freedom linear transformation; (5) structural image segmentation, which divides the structural image into gray matter, white matter and cerebrospinal fluid; (6) nuisance covariates regression, including the Friston-24 motion parameters, white matter signals, cerebrospinal fluid signals and linear drift. Note that the multiple direction motion parameters and the mean frame-wise displacement (FD) of each subject were calculated to evaluate and compare the head motion, results were listed in Table 1 and Supplementary Table 1; (7) based on the segmented images, the individual space was transformed to the MNI space using the DARTEL (diffeomorphic anatomical registration through exponentiated lie algebra) tool, and the resampled voxel size is 3 mm × 3 mm × 3 mm.

**TABLE 1:** Clinical and demographic characteristics of ADHD patients and HCs.

Characteristics	ADHD (*N* = 50)	HC (*N* = 28)	Statistics	*P*-value
Age	8.26 ± 1.93	8.93 ± 1.46	*t* = −1.596	0.115
Gender (male/female)	(43/7)	(20/8)	χ^2^ = 2.454	0.117
Head motion (FD) (mm)	0.105 ± 0.069	0.093 ± 0.054	*t* = 0.791	0.432
Full-scale IQ	116.94 ± 17.45	122.43 ± 13.84	*t* = −1.430	0.157
**Conners’ Parent Rating Scale**				
Conduct problem	1.23 ± 0.51	0.39 ± 0.33	*t* = 8.869	<0.001***
Study problem	1.93 ± 0.68	0.64 ± 0.62	*t* = 8.217	<0.001***
Psychosomatic	0.41 ± 0.35	0.15 ± 0.24	*t* = 3.859	<0.001***
Impulsive–hyperactive	1.70 ± 0.63	0.52 ± 0.50	*t* = 8.441	<0.001***
Anxiety	0.49 ± 0.47	0.35 ± 0.29	*t* = 1.646	0.104
Hyperactivity index	1.62 ± 0.54	0.50 ± 0.40	*t* = 9.457	<0.001***
**Wisconsin Card Sorting Test**				
Perseverative errors	16.20 ± 9.24	12.00 ± 5.55	*t* = 2.506	0.014*
Non-perseverative errors	13.62 ± 6.30	14.75 ± 4.96	*t* = −0.818	0.416

*P < 0.05; ***P < 0.001.

### Dynamic R-fMRI Indices Calculation

R-fMRI indices were calculated with following procedures:

*ALFF* (Zang et al., 2007; Zou et al., 2008). After Fourier transform of time series of a voxel, ALFF takes the mean amplitude of a specific frequency range (0.01–0.08 Hz) as the intensity of local activity of the voxel.

*ReHo* (Zang et al., 2004). The Kendall’s coefficient of concordance between the time series of a given voxel and the time series of its closest voxel.

*DC* (Buckner et al., 2009; Zuo et al., 2012). In this study, we calculate the Pearson correlation coefficient between the time course of each voxel and the time course of every other voxel in the whole brain. For each voxel, DC denotes the sum of connections that showed a correlation coefficient exceeding 0.25 (Buckner et al., 2009; Yan et al., 2017). Correlations below this threshold were considered mainly caused by signal noise. Meanwhile, it has been proven that different threshold selection from 0.1 to 0.3 did not significantly change the network structure (Buckner et al., 2009). The obtained DC values were converted to Z-scores by subtracting the global mean DC and dividing by the SD of the whole brain DC, the obtained voxel-wise DC map.

*VMHC* (Zuo et al., 2010). Function images were transformed into a symmetric space. Then, Pearson’s correlation coefficients between time series from homologous brain regions were calculated (VMHC). Finally, the VMHC maps were transformed by Fisher Z-transformation for subsequent analysis.

*GSC* (Hahamy et al., 2014; Power et al., 2017). BOLD signals from all the voxels were averaged as the global signal. Pearson correlation coefficients between each voxel time course and the global signal were calculated to get the GSC maps, finally The GSC maps included in the subsequent analysis were Fisher Z-transformed.

In particular, dynamics of these R-fMRI indices were analyzed using Temporal Dynamic Analysis (TDA) toolkits based on DPABI (Yan et al., 2016). Specifically, R-fMRI indices mentioned above were computed with the hamming windows (window length = 32 TR, window step = 4 TR, generating 50 windows). Previous studies have revealed that even a window length ranged from 30 to 60 s is able to capture dynamic functional connectivity fluctuations and different window lengths do not yield significantly different results (Preti et al., 2017). Also, previous studies have demonstrated that the step size does not dramatically affect the variance of dynamic properties of R-fMRI (Liao et al., 2019). Therefore, we used a window length of 32 TR (64 s) and a step size of 4 TR (8 s) (Yang et al., 2020). More importantly, different window length (48 TR/64 TR) were used to validate our results (see Supplementary Materials). The R-fMRI indices mentioned above were prefixed with a *d* to indicate the dynamic indices. For example, dALFF represents dynamic ALFF. Further, the standard deviation (SD) of each index across the time series from each voxel was calculated and normalized across the voxels to measure the variability in these dynamic R-fMRI indices. Finally, the normalized SD maps of each dynamic R-fMRI index were smoothed (FWHM = 4 mm) to improve the signal-to-noise ratio.

### Concordance Analysis

The concordance of five dynamic R-fMRI indices (ALFF, ReHo, DC, VMHC, and GSC) were calculated. In specific, volume-wise and voxel-wise concordance was measured by Kendall’s W among these indices (Yan et al., 2017). The volume-wise concordance (spatial concordance) was calculated in two steps. First, compute the Kendall’s W of the five indices across voxels for each window. Second, the dynamic volume-wise concordance was computed as the averaged Kendall’s W across all time windows for each participant. The voxel-wise concordance (temporal concordance) was measured by the Kendall’s W of the five indices across all time windows. By doing this, each voxel (voxel-wise) was assigned a concordance value. These voxel-wise concordance maps were then smoothed (FWHM = 4 mm) for subsequent analyses. Thus, for each participant, they got one volume-wise concordance value and N (*N* = number of voxels) voxel-wise concordance values.

Importantly, we validated the results with the window length of 48 and 64 TR.

### Statistical Analysis

We compared age, IQ, ADHD-relevant clinical variables between ADHD patients and healthy controls using two-sample *t*-tests. Meanwhile, gender distributions were compared using chi-square test. These statistical analyses were performed with SPSS 23.0.

The dynamic R-fMRI indices and voxel-wise concordance were compared using two-sample *t*-test, and the whole-brain gray mask was applied, and the results were corrected by multiple comparisons using Gaussian random field theory (GRF, voxel-wise *p* < 0.001, cluster-wise *p* < 0.05, two-tailed). Meanwhile, the volume-wise concordance was compared using analysis of covariance (ANCOVA). Further, Pearson or Spearman partial correlation was used to quantify the associations between dynamic indices and ADHD-relevant clinical test scores. Note that the age, gender, IQ, and head motions are controlled as covariates.

## Results

### Clinical and Demographic Characteristics

The characteristics of samples were summarized in Table 1. In particular, the ADHD group and the HCs group showed no significant differences in age, gender, head motion, or IQ. As measured by CPRS, ADHD patients exhibited significantly higher hyperactive and impulsive, and had more serious behavior and learning problems. In Wisconsin Card Sorting Test, the number of perseverative errors in ADHD group was higher than that in HC group (*P* = 0.014).

### Alterations of Variability in ADHD

**FIGURE 1:**
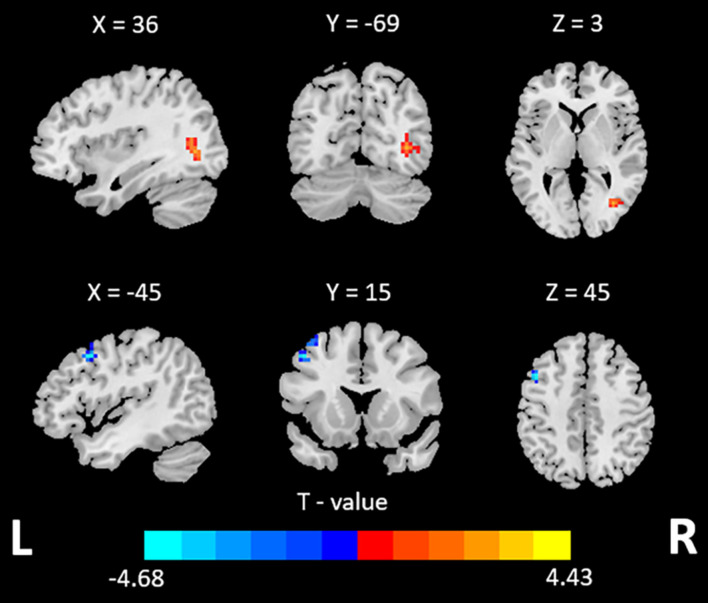
Brain regions showing different dALFF variability between the ADHD and HCs.

Compared with HCs, the ADHD group exhibited increased dALFF variability in right middle occipital gyrus and decreased dALFF variability in left middle frontal gyrus (GRF, voxel-wise *p* < 0.001, cluster-wise *p* < 0.05, two-tailed; see Table 2 and Figure 1). No significant between-group difference was found for the dynamics of other R-fMRI indices.

**TABLE 2:** Brain regions with significant differences in R-fMRI dynamic indices (SD) between ADHD patients and HCs.

Measurements	Brain regions	MNI coordinates (x, y, z)			Voxels	Peak *t*-values	*P*-value (cluster-lever)
dALFF	Right middle occipital gyrus	36	−69	0.3	45	4.43	0.002
	Left middle frontal gyrus	−45	15	45	36	−4.68	0.005

### Alterations of Volume-Wise and Voxel-Wise Concordance in ADHD

**FIGURE 2:**
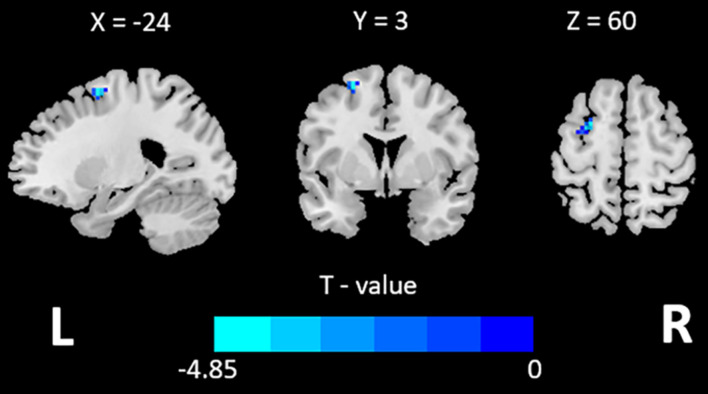
Brain region showing different voxel-wise concordance between the ADHD and HCs.

The ANCOVA results indicated no significant differences in volume-wise concordance between the ADHD group and HC group. However, ADHD patients showed decreased voxel-wise concordance in left middle frontal gyrus (GRF, voxel-wise *p* < 0.001, cluster-wise *p* < 0.05, two-tailed; see Table 3 and Figure 2).

**TABLE 3:** Brain region with decreased voxel-wise concordance in ADHD.

Brain Regions	MNI Coordinates (x, y, z)			Voxels	Peak *t*-values	*P*-value (Cluster-lever)
Left middle frontal gyrus	−24	3	60	40	−4.85	0.002

### Negative Correlation With the Non-perseverative Errors

**FIGURE 3:**
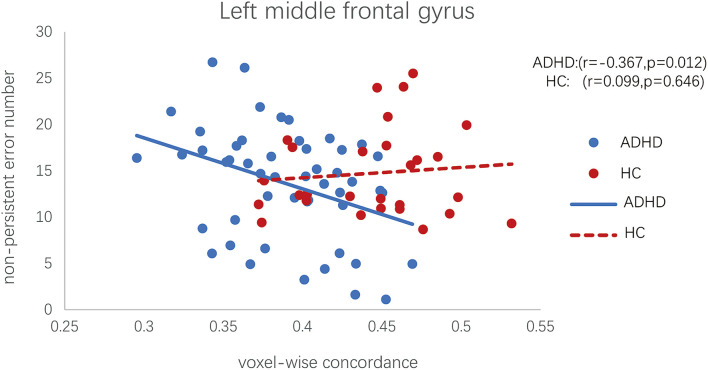
Scatter plot of the voxel-wise concordance in the left middle frontal and the non-perseverative errors in WCST of patients with ADHD. (Pearson partial correlation scatter diagram, controlling for effects of head motion, age, IQ, and gender).

No significant correlations were found between the CPRS scores and altered dALFF variability for the ADHD group. However, the abnormal voxel-wise concordance of the left middle frontal was negatively correlated with the non-perseverative errors in WCST of patients with ADHD (Pearson partial correlation, *r* = −0.367, *p* = 0.012; see Figure 3). Meanwhile, we found no correlation between left middle frontal voxel-wise concordance and non-persistent errors in HC group (Pearson partial correlation, *r* = 0.099, *p* = 0.646; see Figure 3).

### Validation of the Results

The validation results with the window length of 48 and 64 TR showed good agreement with the results mentioned above (Supplementary Tables 2–5 and Supplementary Figures 1–6).

## Discussion

Abnormality in intrinsic brain activities is tightly associated with ADHD. This study systematically compared the dynamic changes in resting-state brain activities of patients with ADHD with HCs. Specifically, we calculated the temporal variability, spatial and temporal concordance of dynamic R-fMRI indices including ALFF, ReHo, DC, VMHC, and GSC in both groups. These properties were then compared between groups and correlated with the clinical variables, in which both the alterations and correlations were identified.

### Altered Variability in Occipital and Frontal Gyrus

Specifically, for the variability, ADHD patients exhibited increased dALFF variability in right middle occipital gyrus and decreased one in left middle frontal gyrus, which may reflect the hyper-activation in visual cortex and hypo-activation in frontal cortex. This is consistent with a previous study in which decreased ReHo was found in frontal and increased ReHo and ALFF was found in visual cortex (An et al., 2013).

The development of the frontal lobe is essential for the acquisition, execution and control of a wide range of functions (Rosch and Mostofsky, 2019). And frontal lobe is a component of the brain region of the cognitive-attention network (cingulate-frontal lobe-parietal lobe), these areas are typically involved in cognitive, attentional, executive function, response inhibition, motor control, and working memory (Bush, 2011). Deficits in frontal lobe can lead to a series of developmental disorders like ADHD, and increase the psychopathological vulnerability. Many studies have revealed that the structural and functional abnormalities in the frontal lobe of patients with ADHD (Albajara Sáenz et al., 2019). For example, the volume of gray matter (Shaw et al., 2011) and the ALFF (Yang et al., 2011) in the middle frontal gyrus was decreased in ADHD patients. One previous study also exhibited decreased functional connectivity between right middle frontal gyrus and whole brain, and the decreased was associated with lower IQ (Shaw and Sudre, 2021). Consistent with the previous studies, we also observed decreased dALFF variability in middle frontal gyrus. The decreased dALFF variability indicated abnormal temporal fluctuations of brain activity in this region. Such an abnormal pattern may underlie the deficit in cognitive control of patients with ADHD, and the failure in cognitive control plays a key role in the main symptoms of ADHD.

Structural and functional alterations in occipital were also reported in previous studies. For example, reduced gray matter volume in the right middle occipital gyrus (Wang L. J. et al., 2020) and hyper-activation of occipital lobe function was found in ADHD (Tian et al., 2008; Icer et al., 2019), indicating that atypical visual processing may play a key role. Previous study also reported that the decreased connectivity between the frontal cortex and visual cortex was associated with increased severity in ADHD symptoms (Wang M. et al., 2020). Meanwhile, the interaction between the dorsal attention network and visual cortex is important for maintaining attention (Shulman et al., 2009). These findings suggest that brain activities in frontal and occipital regions and their interactions may play a key role in ADHD, which awaits further investigation.

### Altered Concordance in Left Middle Frontal Gyrus

Alterations in concordance among the dynamic R-fMRI indices were also identified in patients with ADHD. A recent study found that R-fMRI regional indices was high concordant in brain cortical and subcortical areas across the time window (Yan et al., 2017), and the voxel-wise concordance reflects homogeneity between these indices. Several studies on schizophrenia or MDD have found abnormal voxel-wise concordance in multiple brain regions (Zhu et al., 2018, 2019). These findings suggest that the concordance of dynamic indices may be an important biomarker for neuropsychiatric disorders. In our study, ADHD patients exhibited decreased voxel-wise concordance in the left middle frontal, which was consistent with the decreased dALFF variability in middle frontal gyrus, indicating maladaptive in frontal lobe.

### Correlation Between Concordance of Frontal Gyrus and Executive Function

In addition, we found that the voxel-wise concordance change in left middle frontal gyrus was negatively correlated with non-perseverative errors in WCST of ADHD group. Whereas, we did not find a correlation between them in the HCs. WCST is widely used to assess executive function, including cognitive flexibility, working memory, continuous attention and response inhibition (Godinez et al., 2012). Non-perseverative error measures the difficulties in working memory, sustained attention, and response inhibition (Barceló, 1999; Barceló and Knight, 2002; Godinez et al., 2012). Furthermore, non-perseverative errors have been found to be associated with frontal lobe function in patients with ADHD (Qian et al., 2018). However, no significant difference in non-perseverative errors was found between ADHD patients and healthy controls. This may due to the relatively small sample size, which might induce more variance and undermine the difference between these two groups. Nevertheless, this implies that the concordance in left middle frontal gyrus can be a sensitive biomarker that can capture the changes in non-preservative errors in ADHD patients.

### Clinical Applications

Our findings highlighted the role of frontal lobe in the neuropathological mechanism of ADHD, and demonstrated that concordance of R-fMRI indices can serve as a biomarker to indicate the deficit in frontal lobe. To date, the diagnosis of ADHD is still based on symptom scores from clinical interviews, which can be subjective and can be influenced by many factors, such as the experience, stereotype, and cultural differences. Neuropsychiatric biomarkers can help to build a standard and objective diagnosis system, which can benefit the patients. R-fMRI is among the neuropsychiatric tools that have the potential to find the biomarkers (Abi-Dargham and Horga, 2016). Such biomarkers, combined with the rapidly developed machine learning algorithms, researchers are able to precisely classify the ADHD from healthy control (Du et al., 2018). R-fMRI indices such as ReHo (Wang et al., 2013) and functional connectivity (Fair et al., 2012) have been frequently used by these researchers. Functional concordance incorporates features from multiple metrics, which are fully explored in the current study, can also be used to train the machine learning models, and thereby can promote the accuracy of classification and prediction of ADHD.

Further, the behavioral dysfunction may be too subtle to be measured, just like the non-perseverative errors that failed to be capture by the current study. However, as mentioned above, the R-fMRI indices as proposed by the current study are able to evaluated the maladaptive of brain functions.

### Limitations

This study has several limitations. First, no significant differences were found among some dynamic indices, which may be due to the relatively small sample size. A larger sample size is needed to validate the alterations in dynamic brain function in ADHD and examine the relationship between dynamic variables and psychological data. Second, the current study only focus on the BOLD signal from gray matter. However, previous studies have demonstrated the white matter signals also provide valuable information (Peer et al., 2017; Ding et al., 2018; Li et al., 2020a,b). Hence, further studies are need to explore the altered variability and concordance of white matter signals in patients with ADHD. Third, this study used a variety of indices to reflect different patterns of dynamic spontaneous brain activity, but the seed-based functional connectivity analysis (FC) and independent component analysis (ICA) were not included in the analysis, this is because ICA may be biased by component selection and FC may be biased by seed definition. In future studies, more supplementary R-fMRI indices should be considered, which may help to describe the spontaneous brain activity more thoroughly and understand its underlying physiological processes more deeply.

## Conclusion

The current study investigated the variability and concordance of a range of commonly used R-fMRI indices in ADHD. We found that patients with ADHD exhibited increased dALFF variability in right middle occipital gyrus and decreased dALFF variability in left middle frontal gyrus. Meanwhile, we found that voxel-wise concordance of a series of R-fMRI indices in patients with ADHD was decreased in frontal lobe, and a greater decrease was related to worse WSCT performance. These findings suggest that dynamic analysis and functional concordance may provide new insights into the neuropathological mechanism of ADHD.

## Data Availability Statement

The raw data supporting the conclusions of this article will be made available by the authors, without undue reservation.

## Ethics Statement

The studies involving human participants were reviewed and approved by the Ethics Committee of the First Affiliated Hospital of Wenzhou Medical University. Written informed consent to participate in this study was provided by the participants’ legal guardian/next of kin.

## Author Contributions

FL and JT analyzed the data and wrote the manuscript. RZ analyzed the data. SC and AQ collected the MRI data. CY, XZ, and BC evaluated the subjects. ZH and MW designed and supervised the experiment, and finalized the manuscript. All authors reviewed the manuscript and made significant contribution to this manuscript.

## Conflict of Interest

The authors declare that the research was conducted in the absence of any commercial or financial relationships that could be construed as a potential conflict of interest.

## Publisher’s Note

All claims expressed in this article are solely those of the authors and do not necessarily represent those of their affiliated organizations, or those of the publisher, the editors and the reviewers. Any product that may be evaluated in this article, or claim that may be made by its manufacturer, is not guaranteed or endorsed by the publisher.
