# Supplementary material for: Altered Variability and Concordance of Dynamic Resting-State fMRI Indices in Patients With Attention Deficit Hyperactivity Disorder
Source: Front Neurosci. 2021 Sep 16;15:731596. doi: 10.3389/fnins.2021.731596 (PMC8481633; doi:10.3389/fnins.2021.731596)
Supplement: Supplementary file 1 [file Table_1.docx]

**Supplementary tables**

**Table S1:** Multiple direction motion parameters of ADHD patients and HCs

| Motion parameters | ADHD (N=50) | HC (N=28) | Statistics | P value |
| --- | --- | --- | --- | --- |
| Mean translational (mm)  X | 0.081 (0.055, 0.140) | 0.070 (0.052, 0.119) | Z = -0.677 | 0.498 |
| Y  Z  Mean rotational (°)  Roll  Pitch  Yaw | 0.119 (0.059, 0.184)  0.288 (0.166, 0.501)  0.305 (0.128, 0.461)  0.165 (0.086, 0.300)  0.141 (0.109, 0.254) | 0.093 (0.039, 0.175)  0.320 (0.150, 0.443)  0.215 (0.108, 0.416)  0.106 (0.061, 0.197)  0.124 (0.052, 0.254) | Z = -0.833  Z = -0.208  Z = -1.062  Z = -1.979  Z = -1.490 | 0.405  0.835  0.288  0.048 *  0.136 |

Data are presented as median (p25, p75). Note: P<0.05: *

**Table S2:** Brain regions with significant differences in R-fMRI dynamic indices (SD) between ADHD patients and HCs (window length 48TR).

| Measurements | Brain regions | MNI coordinates (x, y, z) | Voxels | Peak t values | P value  (Cluster-lever) |
| --- | --- | --- | --- | --- | --- |
| dALFF | Right middle occipital gyrus | 36 -69 3 | 43 | 4.43 | 0.002 |
|  | Left middle frontal gyrus | -42 15 42 | 39 | -4.90 | 0.003 |

**Table S3:** Brain region with decreased voxel-wise concordance in ADHD (window length 48TR).

| Brain Regions | MNI Coordinates (x, y, z) | Voxels | Peak t values | P value  (Cluster-lever) |
| --- | --- | --- | --- | --- |
| Left middle frontal gyrus | -27 0 63 | 34 | -4.52 | 0.003 |

**Table S4:** Brain regions with significant differences in R-fMRI dynamic indices (SD) between ADHD patients and HCs (window length 64TR).

| Measurements | Brain regions | MNI coordinates (x, y, z) | Voxels | Peak t values | P value  (Cluster-lever) |
| --- | --- | --- | --- | --- | --- |
| dALFF | Right middle occipital gyrus | 36 -69 3 | 39 | 4.27 | 0.003 |
|  | Left middle frontal gyrus | -42 15 42 | 51 | -5.12 | 0.001 |

**Table S5:** Brain region with decreased voxel-wise concordance in ADHD (window length 64TR)

| Brain Regions | MNI Coordinates (x, y, z) | Voxels | Peak t values | P value  (Cluster-lever) |
| --- | --- | --- | --- | --- |
| Left middle frontal gyrus | -27 0 63 | 32 | -4.40 | 0.003 |

**Supplementary figures**

**Figure S1:** Brain regions showing different dALFF variability between the ADHD and HCs. (window length 48TR).

**
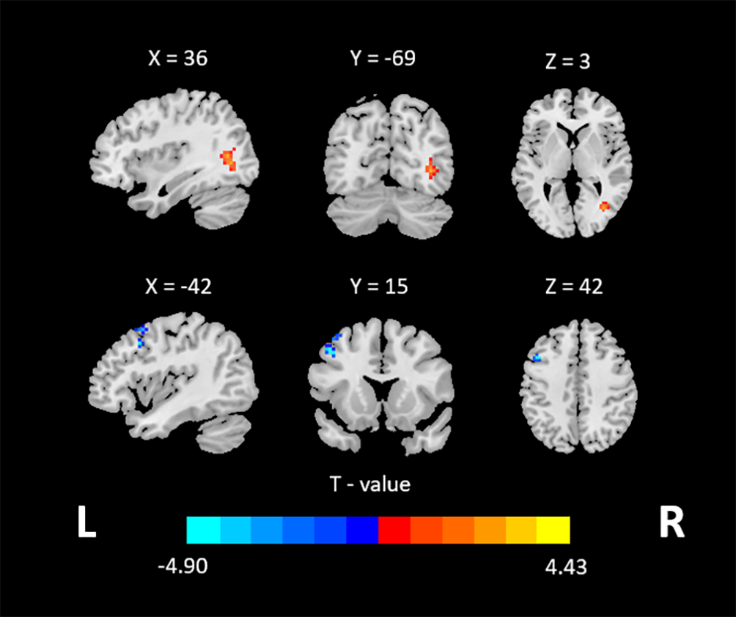
**

**Figure S2:** Brain region showing different voxel-wise concordance between the ADHD and HCs (window length 48TR).

**
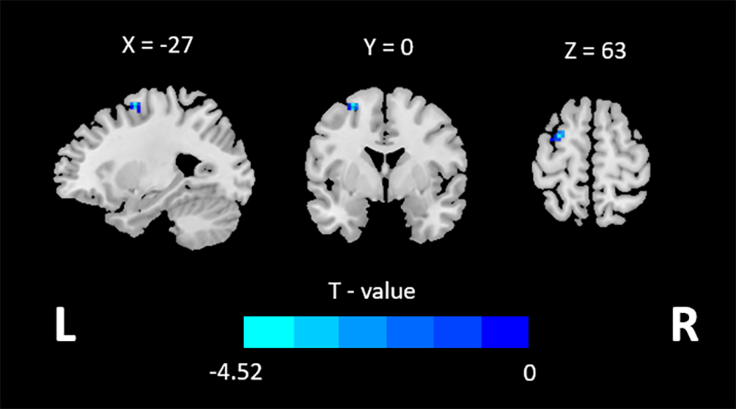
**

**Figure S3** Scatter plot of the voxel-wise concordance in the left middle frontal and the non-perseverative errors in WCST of patients with ADHD (window length 48TR). Pearson partial correlation scatter diagram, controlling for effects of head motion, age, IQ and gender.

**
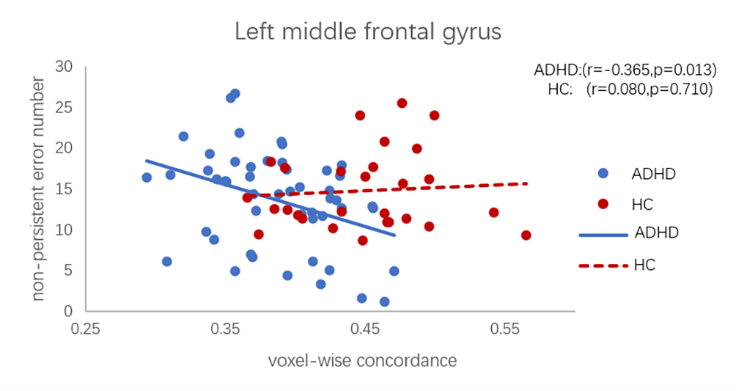
**

**Figure S4** Brain regions showing different dALFF variability between the ADHD and HCs. (window length 64TR).

**
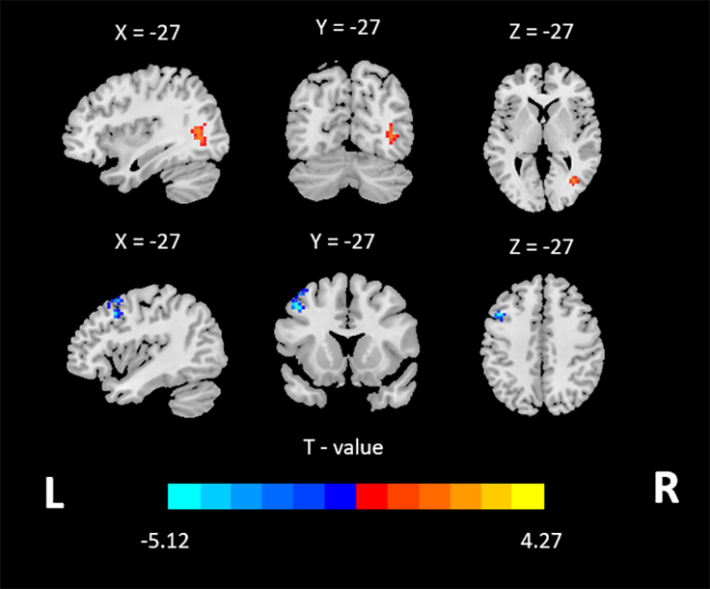
**

**Figure S5** Brain region showing different voxel-wise concordance between the ADHD and HCs (window length 64TR).

**
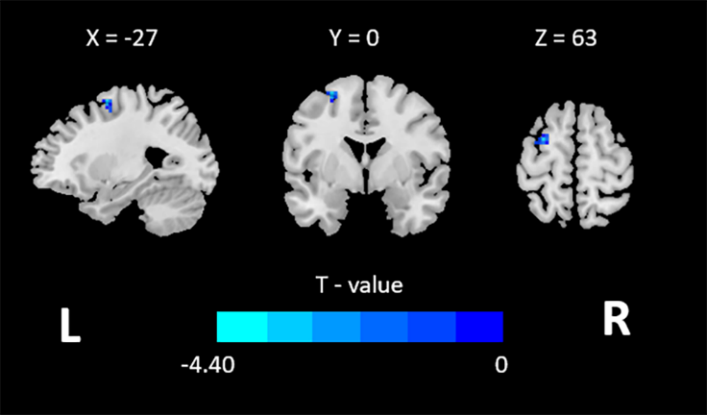
**

**Figure S6** Scatter plot of the voxel-wise concordance in the left middle frontal and the non-perseverative errors in WCST of patients with ADHD (window length 64TR). Pearson partial correlation scatter diagram, controlling for effects of head motion, age, IQ and gender.

**
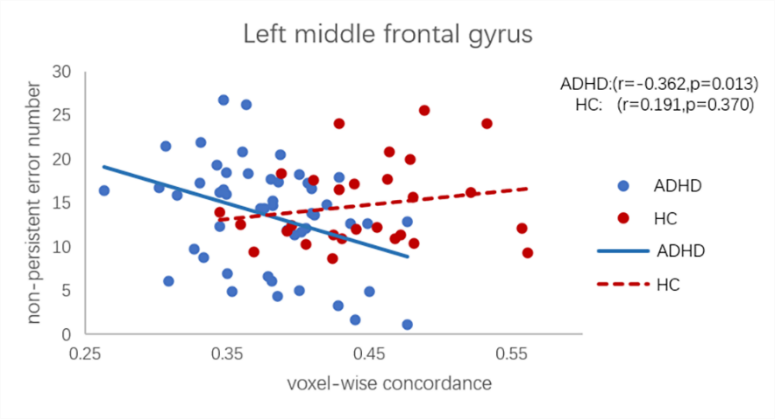
**
